# Technology Use and Preferences for Mobile Phone–Based HIV Prevention and Treatment Among Black Young Men Who Have Sex With Men: Exploratory Research

**DOI:** 10.2196/mhealth.6436

**Published:** 2017-04-13

**Authors:** Ian W Holloway, Terrell JA Winder, Charles Herbert Lea III, Diane Tan, Donte Boyd, David Novak

**Affiliations:** ^1^ Department of Social Welfare Luskin School of Public Affairs University of California Los Angeles, CA United States; ^2^ Department of Sociology University of California Los Angeles, CA United States; ^3^ School of Social Work University of Washington Seattle, WA United States; ^4^ Department of Health Policy and Management Fielding School of Public Health University of California Los Angeles, CA United States; ^5^ OLB Research Institute Online Buddies, Inc. Cambridge, MA United States

**Keywords:** HIV, AIDS, mobile applications, African Americans, men’s health, homosexuality, bisexuality, young adult

## Abstract

**Background:**

Black young men who have sex with men (BYMSM) experience higher human immunodeficiency virus (HIV) incidence than their white and Latino counterparts.

**Objective:**

The aim of our study was to understand BYMSM’s preferences for mobile phone–based HIV prevention and treatment in order to inform culturally tailored interventions to reduce the spread of HIV and improve HIV treatment outcomes in this population.

**Methods:**

Qualitative focus groups (N=6) with BYMSM aged 18-29 years (N=41; 46%, 19/41 HIV-positive) were conducted to elucidate their preferences for the design and delivery of mobile phone–based HIV prevention and treatment interventions. A modified grounded theory approach to data analysis was undertaken using ATLAS.ti textual analysis software.

**Results:**

Participants preferred holistic health interventions that did not focus exclusively on HIV prevention and treatment. Issues of privacy and confidentiality were paramount. Participants preferred functionality that enables discreet connections to culturally competent health educators and treatment providers who can address the range of health and psychosocial concerns faced by BYMSM.

**Conclusions:**

Mobile phone–based HIV prevention has the potential to increase engagement with HIV prevention and treatment resources among BYMSM. For these approaches to be successful, researchers must include BYMSM in the design and creation of these interventions.

## Introduction

### Background and Significance

Although gay, bisexual, and other men who have sex with men (MSM) comprise approximately 2% of the US population, they accounted for 67% of all estimated new human immunodeficiency virus (HIV) infections in 2014 [1]. Black MSM are disproportionately infected with HIV comprising 38% of all HIV diagnoses among gay and bisexual men in 2014, and 78% of diagnoses among all black men in 2014 [2,3]. Black young MSM (BYMSM) between the ages of 13 and 24 years accounted for over half (54%) of new HIV infections among all MSM in that age group [3]. In Los Angeles (LA) County, youth aged 18-29 years constituted 40% of all HIV diagnoses among black men in 2013 [4], and 71% were unaware they were HIV-positive [5]. These data demonstrate that despite current prevention efforts, BYMSM remain disproportionately affected by HIV both nationally and locally.

Previous research indicates that BYMSM face many barriers to HIV testing and treatment [6-8]. In particular, if diagnosed with HIV, BYMSM are less likely to receive appropriate HIV care, be on antiretroviral therapy (ART), or perceive they have access to medication, often due to factors such as economic disadvantage and limited access to culturally appropriate health care [2]. BYMSM also may experience disapproval, discrimination, and homophobia from their families and faith communities and may resist disclosing their sexuality out of fear of rejection [5,7,9]. Such barriers highlight the need to develop tailored HIV prevention and treatment interventions that address the complex structural and psychosocial barriers impacting the health and wellness of BYMSM. Whereas some HIV prevention and treatment interventions target black MSM (ie, Many Men, Many Voices; The Bruthas; MAALES; ES-HIM), few are designed specifically for BYMSM [10-14]. Culturally tailored interventions are especially important when targeting certain subpopulations since varying cultures have been known to espouse different knowledge and attitudes in regards to HIV risk [15]. Nevertheless, previous research demonstrates that culturally appropriate materials can positively impact health behaviors among BYMSM [16].

### Mobile Technology, Young Adults, and MSM

Mobile technology, including the Web and mobile phone apps have become important venues for information seeking, communication, and social networking [17]. Young adults aged 18-29 years in the United States represent a ‟digital generation”: 83% of this population owned a mobile phone and 97% reported using the Web or email through a mobile device as of January 2014 [17]. Young men who have sex with men (YMSM) also use mobile technology to seek sexual health information and find mobile phone–based HIV prevention interventions acceptable [18-20]. In Los Angeles, 89% of racially or ethnically diverse young MSM mobile phone users accessed the Web to find information about HIV or acquired immune deficiency syndrome (AIDS), whereas 79% used the Web to find an HIV testing location [21].

MSM are also increasingly using geosocial networking (GSN) apps such as Grindr, Jack’d, and Scruff to locate sexual partners, meet friends, and connect to gay communities [22,23]. The use of GSN apps among MSM has been associated with an increased risk for sexually transmitted infections (STIs). A large community-based study by Beymer et al [24] found that MSM in Los Angeles who used GSN apps to meet sexual partners had greater odds of testing positive for gonorrhea and chlamydia compared with those who had not used GSN apps to meet sexual partners. Given the widespread use of technology among young adult populations, the associations between GSN app use and HIV or sexually transmitted infection (STI) risk, and YMSM’s willingness to receive HIV prevention information via mobile phone, tailored mobile phone–based prevention and treatment interventions are needed to address HIV-related health disparities among BYMSM [21].

### Mobile Technology Interventions and BYMSM

Previous research has demonstrated the need for and effectiveness of technology-based interventions in improving outreach, testing, and linkage to information about and services for HIV among YMSM [25-27]. Although limited, some studies document the outcomes of technology-based interventions targeting BYMSM. For instance, Hightow-Weidman’s [16] pilot of HealthMpowerment (HMP), a 4-week, interactive, theory-based Web-based HIV and STI intervention targeting black men in North Carolina (aged 18-30 years) found that participants in the intervention group reported increased intentions to use condoms, as well as engagement in preparatory condom use behaviors, compared with control group participants. HMP also maintained a 90% retention rate at one-month following the intervention and a 78% retention rate at 3 months. A Philadelphia-based, 12-week, text messaging (short message service, SMS) HIV prevention intervention targeting sexually active BYMSM (16-20 years old) who owned a mobile phone, sent 3 weekly HIV prevention-related text messages to intervention group participants, and 3 weekly nutrition-related text messages to control group participants. This study found higher awareness of sexual health among intervention group participants compared with the control group participants at follow-up [28].

The development of mobile phone app interventions for BYMSM has the potential to increase HIV testing and treatment among this high-risk population. Technology-based interventions offer opportunities to engage MSM who are less accessible to researchers and clinicians due to stigma associated with sexual minority identity disclosure, yet who may still engage in high-risk behaviors [29]. This type of intervention is promising because it could be accessed privately by users, helping to circumvent the stigma associated with same sex-sexual behavior or HIV-positive status among some BYMSM [30]. YMSM participating in technology-based HIV prevention interventions and formative research on mobile technology also express satisfaction with and willingness to use mobile phone–based HIV prevention programs [16,21,26]. However, few HIV prevention interventions have been tailored for mobile phone platforms and many fail to attract mobile phone users, receive low user ratings, and are not commonly designed for racially or ethnically diverse populations [2].

### Present Study

Given increasing HIV rates among BYMSM, the psychosocial barriers they face related to HIV testing and treatment and their access to and use of mobile phones apps, there is a critical need and opportunity to develop innovative, culturally tailored mobile phone–based HIV prevention strategies for this population. However, little research has qualitatively examined BYMSM’s use of apps and preferences for mobile phone–based HIV prevention and treatment, which may be helpful in understanding how to tailor mobile phone–based HIV prevention and treatment efforts for BYMSM. Using qualitative methods, this study sought to elucidate the types and patterns of technology use among a sample of BYMSM in order to better understand their preferences for mobile phone app interventions as a mechanism to promote outreach, HIV testing, and linkage to appropriate HIV and health-related services.

## Methods

### Participants and Procedures

Data were gathered through focus groups with BYMSM in Los Angeles, California (6 focus groups, 6-8 participants per group, N=41). For this study, we defined youth according to Arnett’s (2000) definition of emerging adulthood [31]. Participants were recruited through flyers posted in a range of community-based agencies serving BYMSM throughout LA County. Flyers instructed potential participants to call a central number where they were screened for eligibility. Eligible BYMSM were 18-29 years old; identified as male; identified as black; identified as gay, bisexual, or had had sex with a man in the past 6 months; and had access to a mobile phone. A total of 49 potential participants inquired about the study, 3 were ineligible, and 5 screened eligible but did not attend their scheduled group.

Each focus group lasted 90-120 minutes, was audio-recorded, and conducted in English in private rooms at 2 community-based agencies serving BYMSM in Los Angeles. Prior to the initiation of focus groups participants completed a pre-focus group assessment that asked them to provide information about their demographic characteristics, mobile phone ownership, and social media use. Two trained members of the research team led each focus group and another took notes. Participants were assigned identification numbers, which were used to protect their identities and to track their responses throughout the focus group discussions. Each participant was paid US $20 as an incentive. Study protocols, including informed consent procedures, were approved by the North Campus Institutional Review Board at the University of California, Los Angeles.

### Measures

Semistructured focus group methodology was chosen to enable participants to interact with each other in response to a series of a priori questions developed by the research team. This approach was chosen to generate a richness of data not always possible with individual interviews in addition to being a more efficient way of resolving any seemingly conflicting information [32]. Semistructured focus group guides contained a range of questions regarding technology use, health and wellness, access to community resources, experiences on GSN apps, and desired features for mobile phone–based HIV prevention and treatment interventions. Questions were asked in such a way as to allow participants to discuss the positive and negative aspects of the technologies in question.

For this analysis, we focused on the following 2 semistructured interview topics: (1) experiences and impressions of existing mobile apps for gay and bisexual men, and (2) preferences for functions of a mobile phone app for HIV prevention and treatment targeting BYMSM. First, BYMSM were asked about their current experiences with mobile phone apps and the strengths and weaknesses of existing apps. Specifically, we asked which GSN apps participants preferred and the circumstances under which they used these apps. We asked participants to describe preferred features and functions on existing apps. We were particularly interested in what BYMSM might want to see on a new mobile phone app for promoting HIV prevention and treatment. Finally, participants were asked to consider themselves app designers and to suggest layouts, features, functions, and content areas for a new app.

### Data Analysis

Audio recordings of the focus groups were transcribed verbatim for analysis by an independent agency and reviewed by the research team for accuracy by listening to the audio recordings and comparing them with the written transcripts. Transcripts were then analyzed using a methodology of “coding consensus, cooccurrence, and comparison” [33]. This methodology relies on iterative coding of focus group transcripts using a priori and emergent codes and team meetings to refine analytic codes and establish interrater reliability. Specifically, the research team reviewed an initial sample of focus group transcripts to identify key themes via in vivo coding, which formed the basis of a formal codebook. The codebook was refined after an iterative coding process and, once finalized, two members of the research team were responsible for coding the interviews separately. Communication between research team members took place after formal coding of all focus groups, for which we used ATLAS.ti textual analysis software [34]. Interrater reliability was calculated by comparing a subset of transcripts and calculating the percent match of a priori codes between the two research assistants. High interrater reliability was achieved (93%). When inconsistencies between coders occurred, a third member of the research team was consulted to discuss and help resolve any inconsistencies. Our analysis focused on the narratives that emerged from discussing the two thematic areas named above (ie, mobile phone app usage patterns and preferences for mobile phone app development).

Analysis was guided by a modified grounded theory approach (ie, theory derived from data and then illustrated by characteristic examples of data) [35]; transcripts were reviewed and memos were written to document initial concepts and to define the boundaries of specific concepts [36]. Field notes and interview transcripts were then independently coded to condense the data into analyzable units. Segments of text ranging from a phrase to several paragraphs were assigned codes based on a priori definitions (ie, from the interview guide) or emergent themes (also known as open coding) [35]. Based on these codes, the computer program ATLAS.ti was used to generate lists of codes, which were then summarized and entered into data matrices (with the focus group on the column and content area on the row) for comparison across focus groups. Through the process of constant comparison, the codes were further condensed into broad themes [35]. In the reporting of results, pseudonyms were developed for each participant to maintain confidentiality and protect the identities of human subjects.

## Results

### Descriptive Statistics

The total sample consisted of 41 BYMSM. Participants’ ages ranged from 19-29 years; the average age being 26 years. More than three-quarters of the sample identified as gay (31/41). Over half had received some education past high school (56%, 23/41) and an additional 27% (11/41) had graduated college. Participants were stably housed: over half (60%; n=24) rented a house or apartment and nearly two-thirds (65%, 33/41) lived with others. More than three-quarters of the sample had part- or full-time employment (78%, 32/41) and more than a third made less than US $12,000 annually.

### Principal Results

The majority of participants used either an Android mobile phone (61%, 25/41) or iPhone (46%, 19/41), which they reported using to communicate most frequently with either friends or romantic partners (59%, 24/41 and 22%, 9/41). The most popular methods of communication via mobile phone were talking (83%, 34/41) and texting (90%, 37/41). Popular social networking sites included Facebook (85%, 34/41), Instagram (85%, 34/41), and Twitter (48%, 19/41). The majority of the sample used social networking apps, with 71% (27/41) reporting daily use. Popular GSN apps for partner seeking included Jack’d (53%, 21/41) and Grindr (18%, 7/41); popular websites for social or sexual networking included Craigslist (38%, 15/41) and Adam4Adam (23%, 9/41). Tables 1 and 2 provide additional information on demographic characteristics and mobile phone and other technology use.

**Table 1:** Demographic characteristics among BYMSM in Los Angeles, California (N=41).

Characteristic		n (%)
Mean Age (in years)		25.8 (3.1)
**Sexual orientation, n (%)^a^**		
	Heterosexual	1 (3)
	Bisexual	7 (18)
	Gay or homosexual	31 (78)
	Other	1 (3)
**Highest educational attainment, n (%)**		
	High school graduate or less	7 (17)
	Some college or trade school training	23 (56)
	College graduate or above	11 (27)
**Current living situation, n (%)^a^**		
	Own home or condo	3 (8)
	Rent house or apartment	24 (60)
	Family member’s house	9 (23)
	Friend’s house, condo, or apartment	1 (3)
	Spouse or lover or sexual partner’s house, condo, or apartment	1 (3)
	Homeless shelter or “safe house”	1 (3)
	Other	1 (3)
**Currently lives with (check all that apply), n (%)^b^**		
	Alone, no other person	8 (20)
	A spouse or lover	5 (13)
	A sexual partner (not spouse)	2 (5)
	Other adult family members	12 (30)
	Close friends or roommates	14 (35)
	Children under the age of 18 years	1 (3)
	Other	1 (3)
**Main source of income in past 6 months (check all that apply), n (%)^b^**		
	Employment (part- or full-time)	32 (78)
	Food stamps, welfare, disability, unemployment	13 (32)
	Other	3 (7)
**Money in the last 30 days, n (%)^a^**		
	Less than US $50 (eg, less than US $600 per annum)	3 (8)
	US $51-US $249 (eg, US $600-US $2999 per annum)	4 (10)
	US $250-US $499 (eg, US $3000-US $5999 per annum)	8 (20)
	US $500-US $999 (eg, US $6000-US $11,999 per annum)	5 (13)
	US $1000-US $2999 (eg, US $12,000-US $35,000 per annum)	9 (23)
	US $3000-US $4999 (eg, US $36,000-US $59,000 per annum)	5 (13)
	US $5000-US $6249 (eg, US $60,000-US $74,999 per annum)	1 (3)
	Refused	5 (13)

^a^Percentage may not equal 100 due to rounding.

^b^Response options included check all that apply; percentages will not add to 100.

**Table 2:** Mobile phone and other technology use characteristics among BYMSM in Los Angeles, California (N=41).

Characteristics		n (%)
**Mobile phone type (check all that apply)^a^**		
	Android	25 (61)
	iPhone	19 (46)
	Other	1 (2)
**Via mobile phone, communicates most with (check all that apply)^a^**		
	Friends	24 (59)
	Spouse or lover	9 (22)
	Casual sexual partner	3 (7)
	Exchange sexual partner (for sex work)	1 (2)
	Roommate	4 (10)
	Family of origin	8 (20)
	Refused	2 (5)
**Uses mobile phone to connect with people by (check all that apply)^a^**		
	Talking	34 (83)
	Texting	37 (90)
	Email	28 (68)
	Apps	31 (76)
	Websites	19 (46)
	Other	1 (2)
**Other devices used (check all that apply)^a^**		
	Laptop	29 (73)
	Desktop computer	10 (25)
	Tablet	15 (38)
	Public computer (eg, at a library)	7 (18)
	Wearable device (eg, Fitbit, Up Band, Nike Fuel Band)	1 (3)
**Social networks used (check all that apply)^a^**		
	Facebook	34 (85)
	Twitter	19 (48)
	Instagram	34 (85)
	Pinterest	3 (8)
	LinkedIn	8 (20)
	Google+	11 (28)
	Snapchat	5 (13)
	MySpace	2 (5)
**Dating or hook-up websites currently used (check all that apply)^a^**		
	Craigslist.org	15 (38)
	Gay.com	2 (5)
	Adam4Adam.com	9 (23)
	Blackgaychat.com	1 (3)
	Match.com	1 (3)
	Other	7 (18)
**Dating or hook-up apps currently used (check all that apply)^a^**		
	Grindr	7 (18)
	Jack’d	21 (53)
	Other	3 (8)
**Frequency of social networking app use to connect with people^b^**		
	Daily	27 (71)
	Weekly	8 (21)
	Monthly	1 (3)
	Less than once per month	1 (3)
	Refused	1 (3)
**Typical way of connecting with people in their life (check all that apply)^a^**		
	In person	24 (60)
	By mobile phone (not mobile phone)	6 (15)
	By mobile phone	31 (78)
	By computer (laptop or desktop)	8 (20)
	By tablet	1 (3)

^a^Response options included check all that apply; percentages will not add to 100.

^b^Percentage may not equal 100 due to rounding.

### Mobile phone App Usage Patterns

As discussed previously, BYMSM used their mobile phones for varied reasons, including communicating with friends and family, checking emails, engaging with social media, and for personal entertainment. Many engaged in a combination of these activities and found that it made them more “efficient.” For example, 29-year-old Darrius used his Android phone to increase his organization and productivity, which he preferred over a regular cellular device:

To be efficient in time, like time management. I love Google Now. Navigating purposes, as far as the public transportation, and driving are key for me...And it’s like everything on social media and the communication aspects—I can use a regular phone for that, you know what I'm saying? But a mobile phone helps you be efficient.

Others found that syncing their apps made their lives easier. The integration of multiple apps was important for Alex, a 28-year-old iPhone user:

What I like most about the Nike+ Running app is the fact that it integrates with the music that’s on my phone, so I’m able to play music as I’m jogging or as I'm running. And then it will adapt—or I can control the music on the go and I don’t have to completely go back into iTunes to adjust. It also simultaneously will tell me distance, location, and pace. So I like that it reminds me every so often what my pace is and what I'm doing without me having to think about it.

When asked why they used their mobile phones and which apps they used most frequently, BYMSM did not mention partner-seeking apps. Whereas this was true across all focus groups, when probed on their use of these apps, almost all BYMSM in the study mentioned using GSN apps such as Grindr, Tinder, and Jack’d. Many mentioned regular use of these apps, while others reported more sporadic use. Some described themselves as “chronic” users. For instance, Marcus, a 23-year-old iPhone user, went so far as to say he was addicted to one:

I’m a chronic (GSN app) user. I’m very addicted to (it) at this point. Since I’ve redownloaded it about 6 months ago, I’ve accumulated about 480 unread messages and I pick and choose who I want to reply to. Just seeing that number, it does something to my self-esteem that I specifically can’t do for myself.

Interactions on these apps were not always positive, however, and many participants experienced racist or derogatory remarks from other users. Marcus went on to share his negative experiences on a popular partner-seeking app:

I would be approached or messaged by Caucasian men and they would make reference to my “big black cock,” of which I don’t have a picture of anywhere on my profile. I felt like they were putting me in the stereotype of big black men that have these knee-dangling penises and they’re just letting everybody suck it. Also one (older) Caucasian man wanted me to be his “slave”...his “bedroom slave.” One man was a Latino. He just wanted to tell me I was a nigger and cussed me out in Spanish, called me “mayate”—that’s Spanish for a bug.

In response to and in anticipation of overtly racist interactions, several participants used app features that blocked racist or aggressive users, filtered for their preferences (in order to avoid other users based on race), or created detailed profiles outlining expectations for communication in order to avoid negative conversations. This allowed participants to “filter by race,” meaning that they would use the filter function to view only other black men and to structure potential partners on the app to a group of users they thought reflected their own desires and responded to them in a manner that they appreciated.

Julian, a 25-year-old iPhone user, shared:

...I have to be honest, you know, I try as much as I can personally to filter through race before I actually meet somebody.

While filtering and blocking were strategies used by many BYMSM across different apps, specific features of a popular app allowed for users to see who had viewed their profile. This in turn allowed them to see the types of people they attracted and decide with whom to speak. Additionally, many participants commented on the detailed nature of many other users’ profiles that stated “what kinds of people they’re interested in,” allowing them to know if a fruitful conversation was probable. As Jeffrey, a 20-year-old Android user, and Nathan, a 21-year-old Android user, discussed:

With who’s been viewing you, you can see how many times they usually reply to people, what kinds of people they're interested in, like black, Latino, bears, strictly friends or something like that...who you message and who you talk to.

Jeffrey

I guess I like how the profile is on (GSN app name) because everything is right there. You have your pictures on one side and then you have all the information about the person on the other side. You can determine if you think you're going to like the person right off the bat because all of the person’s qualities or features is right there.

Nathan

In general, participants appreciated easily accessible information about potential partners and getting tailored feedback on their app use to facilitate partner seeking and limit negative interactions.

### Preferences for Mobile Phone App Development

There was a high degree of support for a mobile phone app for promoting HIV prevention and treatment. However, there was nearly universal agreement that this app should go beyond HIV and address health and wellness more broadly. Many thought that app users should have direct access to trusted doctors and clinicians via an app; yet, participants were torn between whether they should be able to communicate with other users. Some thought that this could be a potential for meeting new friends or even new partners who are also concerned with taking care of their health. Kevin, a 29-year-old Android user commented:

This app should have something so you can talk (to) other people who have the app. For example, he may not want to go to the clinic by himself and maybe someone else is going to go, so maybe they can go (together) (sic)...You know, it should be a message board.

Dejuan, a 20-year-old iPhone user elaborated:

I would say direct (messaging) too. You can meet people that have this app, so (that) you know either this person is protected, they know what they’re doing, or they’re taking care of themself health wise.

Additionally, participants thought it useful to meet someone who has been through the same issues they might be going through and viewed an app as an opportunity to obtain peer advice rather than strictly medical or professional insight.

Elijah, a 21-year-old iPhone user said:

You can actually go to other people for advice. Say, for instance, they already dealt with the problem that you're having, so you can go to them and be like, ‘Oh, what did you do? What stuff did you take?’ And it can actually help you through it.

Some expanded on this idea of using an app to meet people with whom they have shared experiences by looking for a “sponsor.” Ryan, a 29-year-old iPhone user thought that using a mobile phone app to target BYMSM who are newly HIV-positive would provide an avenue for users to connect with someone who could walk them through the HIV health care process:

If you’re targeting young, black, African-American males who are just finding out that they are HIV-positive or afraid to deal with the stigma of being HIV-positive, I think it would be great if you were able to click on an app to find a sponsor or someone that would help guide you through the motions. Tell you, ‘Let’s go find a testing site. I’ll walk you through this.

Conversely, some participants thought that being able to communicate with other users would be a potential breach of the app’s confidentiality and strongly opposed communicating with other users on the platform. These participants thought that you should only be able to connect to a local health provider for services or information. Jordan, a 29-year-old Android user elaborated:

I agree with only doctors, especially in this lifestyle, you know, people are going to say you’ve been doing something else. You don’t want that on the app because that’s going to make people not want to get on the app no more.

Ryan, a 29-year-old iPhone user, further reflected on the importance of confidentiality:

As I said earlier when it comes to apps, everybody knows everybody, knows everybody. And especially when you deal with African-American homosexuals in this environment of West Hollywood...If I choose to go to a testing facility or go somewhere, I’m going to be selective of where I go specifically, so my information doesn’t get to someone’s friend who knows someone who knows someone, (when) it should be confidential.

Participants viewed an app as a way to directly connect to a provider privately and confidentially and as a way to avoid automated machines or culturally insensitive HIV health care providers that could taint their experiences of engagement with HIV prevention and care.

Terrance, a 22-year-old iPhone user stated:

Just to be able to go through that app to make an appointment. I don’t want to have to call; you know what I mean? If I do have to call, then yeah, the number should be on the iPhone, you can click the number and it just directly calls. But on the app, if I’m able to, type, ‘Hey, this spot is open, can (I) come in at this time?’ it saves me talking to Susie at the front desk and the automated service, which both will piss me off.

While the majority of participants were excited about the prospect of a new health app geared toward black gay men, they struggled with whether it should be directed only to BYMSM. Citing the many health disparities that exist more generally for men of color, and particularly black men, some thought that the app would be better if it were general in its approach, but allowed for the user to personalize its content. Deshawn, a 28-year-old iPhone user, thought strongly that the app needed to focus on issues outside of HIV and STDs and to be more encompassing to black men’s health more generally:

Even though we’re African American gay men, we’re still African American men and we still experience the same health disparities that black men in general experience, but we’re so caught up on HIV and STDs that we don’t talk about other stuff like diabetes or colon cancer and all these other things. So I think making it broader will not only expose other people to this, but will also expose us to things.

Furthermore, some participants thought that the app could more broadly cater to the larger black community. Sebastian, a 25-year-old Android user argued:

Don’t just make it a gay thing...Make sure it’s health, period, for black people, because that’s the struggle of trying to get black people in health care.

Despite overall enthusiasm for the creation of an app that would facilitate health and wellness among BYMSM, many participants cited potential barriers to its effectiveness. One such barrier would be if the app were viewed as too “pushy” or forced HIV-related content. Sebastian, contended:

Having too much awareness, like having all the ads about HIV and all the ads about health care, sometimes they make you scared to go. You know, like, people try to push religion on you? It makes you feel like—someone trying to push health care on you.

Other barriers included the accessibility and readability of the information on these apps. For example, participants were concerned about the technical language used on some health websites, such as WebMD. Marcus, a 23-year old iPhone user, stated:

Yeah, I’ve tried WebMD but it wasn’t easy for me to use...I think it was the terminology that I didn’t understand. When I would click on a certain body part and it’d give me this list of stuff, I’m like, ‘What's a tracheal tube?’ you know?

Despite concerns about the use of technical language within the app and fears about privacy and confidentiality, the general consensus among participants was an excitement for the creation of a health app for BYMSM. In order to further refine how an app to promote HIV prevention and treatment for BYMSM might be most successful, participants suggested many features and functions that they would appreciate (see Tables 3 and 4).

**Table 3:** Content for a health app suggested by BYMSM in Los Angeles, CA (N=41).

Content	Example quotes
Rating and reviews for local clinics and providers	Cedric: “I think a Yelp approach...So I think something like that where you can rate the clinic, where you can rate the provider, and be able to give as detailed or as little of information and feedback...”
Statistics and health news	Dejuan: “I think it should have—you know how they have trending topics, Twitter and all that, it should have statistics and stuff like that too, so people can also be aware as well of other things. Like this many people this, or this many people that. This many people came in this day and got this.”
Content on fitness and nutrition	Deshawn: “This may be obvious but I was thinking having the site broken up into clear categories, so mental health versus sexual health versus fitness and nutrition. And then having subcategories under each of those. So from the home page, it’s very easy to access whatever specific information you want. You don’t have to search through the app to find it.”
Other holistic health information not limited to HIV and STDs	Craig: “I think also it would be great to have something that also offers culturally competent mental health services and stuff like that, because beyond just HIV, STD stuff, because there are so many other things that people don't realize that they can have access to resource-wise...”
Localized content about health and upcoming events for BYMSM	Jordan: “Yeah, if you want information, you can login to that app and be like, okay, this is this. Even, it can be different party sites, different clubs going on for the—like, all types of stuff. It’s just an app that makes you want to login because you don’t know what type of information you're going to get.”
Symptom checker	Rohan: “You can have STD information, kind of like how WebMD has it. You can put in symptoms and stuff.”

**Table 4:** Features and functions of a health app suggested by BYMSM in Los Angeles, CA (N=41).

Features and Functions	Example quotes
Direct messaging to clinics and primary care providers	Jaron: “So maybe to be able to be in contact with somebody, like a nurse hotline or something, somebody who could respond to you directly via chat.”
User chat and message boards	Kevin: “You know, it should be a message board. Dejuan: I would say direct too. You can meet friends on here but you only meet people that have this app, so you know either this person is protected, they know what they're doing, they're taking care of themself (*sic*) health wise.”
Geolocation of testing and care clinic locations	Jeffrey: “Yeah, like the tabs, like different tabs. Because my idea was that it would give updates so that could be one tab is the updates. And then the second tab could be, like, a proximity for where the closest clinics are or something like that. And then another tab could be...whatever else we were coming up with. But different tabs so you can swipe through all your stuff.”
Contests and prizes for participating in health games	Nathan: “You can also do—just ask questions about—it can be a random question about, “What is HIV?” and then the first person to answer the question gets movie tickets.”
Find a sponsor for newly diagnosed or out-of-care HIV-positive men	Ryan: “When you think about Grindr, Jack’d, and we talk about confidentiality and the good parts about that is picking and choosing who you can deal with and who you want to and you can block people. I think it would be great if you treat the app just like an AA sponsor. If you're targeting young black, African-American males who are just finding out that they are HIV-positive or afraid to deal with the stigma of being HIV-positive, all the above, I think it would be great if you were able to click on an app to find a sponsor or someone that you can disclose confidentiality to that would help guide you through the motions.”
Integration with popular dating and relationship apps	Devon: “I think maybe having an app that is cater—we know that as, you know, young black men, everybody’s out there having sex. Why not cater to an app that at the forefront is health, where you know that they're having sex, you know what I mean? Instead of something like Jack’d where it’s just a sex site. A4A is just a sex site. That it has health advertisement. How about having something that is a health site that you can find somebody that you can have sex with?”

## Discussion

### Principal Findings

This study was among the first to qualitatively explore BYMSM’s preferences for mobile phone–based HIV prevention and treatment. BYMSM continue to be disproportionately impacted by HIV despite existing prevention efforts [2,3,37]. While mobile technologies represent powerful tools to recruit, engage, and deliver HIV prevention and treatment information to young people [38], few studies have effectively harnessed these technologies to reach BYMSM [39]. Our study sought to understand the ways in which BYMSM engage with technology and offer suggestions to researchers and practitioners who seek to use mobile phone apps to engage this population.

BYMSM in our study were avid mobile phone users who relied heavily on apps in their daily lives for productivity, entertainment, information seeking, and meeting sexual partners. These data are consistent with national trends, which suggest that people of color are the fastest growing user base for mobile phones [40], and other research supporting widespread mobile phone use among MSM [41]. The popularity of mobile phones among BYMSM presents a unique opportunity for HIV prevention and treatment intervention researchers and practitioners. However, a recent study by Muessig and colleagues [39] found 55 existing apps for Android or iPhone as of 2012 aimed at HIV prevention and care. The majority of these apps were infrequently downloaded and not highly rated by consumers, raising questions about the relevance of these kinds of apps. It is important to mention that health apps are often available but that the use of these apps may not necessarily be reported in peer-reviewed journals. That said, the data available in the academic literature suggest a potential disconnect between the needs of consumers and the design and functionality of HIV prevention and treatment apps.

BYMSM in our study were interested in an app for health and well-being, not necessarily one targeted exclusively at HIV prevention and care. Privacy and confidentiality were paramount for our participants, who expressed hesitancy about downloading an app onto their mobile phone that might indicate to others that they were HIV-positive or engaging in behavior that could put them at risk for HIV. Other research has highlighted attention to privacy and confidentiality with BYMSM who may be especially vulnerable to stigma and discrimination for engaging in same-sex sexual behaviors [32]. Participants in our study expressed clearly that in order for an app to be trusted among BYMSM, it would need safeguards in place for personal health information. Security breaches are a major barrier for many technology users—this may be especially true among black MSM, where medical mistrust is well documented [42].

Although participants agreed HIV should not be the sole focus of the app, they were strong advocates of being able to access information about HIV prevention and treatment. Participants liked the idea of using GPS to find nearby testing clinics. Furthermore, participants wanted to read reviews of other users’ experiences at HIV testing and clinical sites, book appointments, and speak to clinical providers on the Web. These functions are already being incorporated into some apps designed for sexual health. Healthvana, for example, is an HIV and STD testing app that allows users to find nearby clinics, read user reviews, see hours of operation and, in some cases, schedule appointments. Healthvana also enables users to request their test results via the app in order to share those results with potential sexual partners. The ability of users to communicate directly with HIV health care providers may present challenges in terms of cost and scalability of health apps. The most successful mobile phone interventions to date use low-cost text messaging for appointment and medication adherence [43-45]. However, collaborations with county health departments that enable a peer health educator or nurse practitioner to be “on call” for messaging with app users could be highly effective at engaging users and directing them to appropriate HIV prevention and treatment services.

Many participants liked the idea of incorporating social media into an app and suggested this as a way to build social support networks with others. Some studies have used existing social media platforms, like Facebook, to deliver HIV-prevention interventions for youth [46,47] and for black MSM [48]. Harnessing Online Peer Education (HOPE) is a Facebook-delivered intervention for black and Latino MSM that assigns participants to private Facebook groups facilitated by peer educators who disseminate information about HIV and engage group members in conversations about safer sex behaviors. In a pilot randomized controlled trial in Los Angeles, network ties among users increased over a 12-week period, which was associated with greater requests for HIV home testing kits and lower self-reported HIV risk behaviors [49]. These findings support the integration of social media functionality in mobile phone apps to increase engagement in HIV prevention and treatment.

While social media functionality that enables users to communicate via message board postings as well as instant message may increase interest in app use, it also presents potential challenges. First, a critical mass of users is necessary to entice potential users to join existing networks. Our participants suggested offering incentives to join and engage with the app in addition to partnerships with local businesses to engage the community through advertising and events. Second, some participants were concerned that social media functionality would facilitate sexual partnership formation on the app. Whereas sexual partner seeking via an app designed with health in mind could lead to safer sexual behaviors among users, participants highlighted that there were already many apps designed for this purpose and expressed interest in integration between these apps and a health app for BYMSM. Finally, some participants worried that by enabling users to talk to one another the risk of unwanted disclosures regarding same-sex sexual behaviors or HIV serostatus would be more likely.

Participants were supportive of an app tailored specifically for BYMSM as it had the potential to offer a Web-based space without the overt and covert racism they often experience on other apps. Discrimination and stigma via social media have been reported by BYMSM qualitatively [50], but have rarely been explored in relation to sexual health for this population. Our participants described strategies for mitigating racism, which seemed to have important implications for the construction of sexual networks established via social media. For example, filtering by race in order to avoid overt race-based hostility or sexual objectification may limit the sexual networks of BYMSM. One hypothesis for increasing rates of HIV infection among BYMSM, despite lower reports of behavioral risk factors (eg, unprotected anal intercourse, substance use), focuses on same race sexual networks, where disease transmission risk is higher due to higher HIV prevalence among BYMSM compared with other racial or ethnic groups [37,51]. Further attention to the ways technology influences sexual network structure and composition, including research to elucidate how technology-based sexual networks are formed and maintained among BYMSM, is needed. While an app exclusively for BYMSM has the potential to reinforce sexual partnering by race, a focus on coping with and responding to racism, homophobia, and other forms of oppression on the Web could be useful to BYMSM.

Technology-based HIV intervention research with MSM has recently emerged with promising results [33,38,49]. To our knowledge, only one published study has specifically targeted BYMSM: Hightow-Weidman and colleagues [38] pilot tested a mobile phone optimized intervention called HealthMPowerment.org with 15 young black MSM and transgender women who have sex with men in North Carolina. Although the sample size was too small to detect statistically significant effects, participants described satisfaction with the intervention, which uses gaming theory as a way to engage participants with the app. Upon conclusion of the 4-week trial, participants described how HMP led to changes in sexual risk behaviors, including increased condom use and HIV testing. This early work demonstrates the feasibility and acceptability of app-based HIV prevention interventions and the potential utility of gaming theory as a framework for engaging BYMSM. Participants in our study also suggested the use of incentives to maintain participant engagement but suggested broadening the app framework beyond HIV prevention to incorporate a more holistic conception of health. Our previous work with the House and Ball communities in Los Angeles demonstrates the many other psychosocial concerns facing black MSM: low educational attainment, unemployment, homelessness, stigma, and discrimination [52]. Attention to these concerns within app platforms may improve the engagement and utility of mobile phone apps designed to improve HIV prevention and treatment.

### Limitations

This qualitative study focused on the experiences of BYMSM using mobile phones in Los Angeles, California. Selection bias introduced by convenience sampling is a limitation and findings may not be representative of all BYMSM. BYMSM were not asked to provide any identifying information during focus groups; however, participation in a group where others might recognize them or disclose their identity may have prevented some potential participants from volunteering to participate. In order to limit biases introduced during coding and thematic analysis, research assistants wrote statements of reflexivity, which increase objectivity in qualitative research.

In our analyses there did not appear to be differences in app use and app preferences by HIV serostatus. This may be due to the fact that we used the same semistructured interview guide for both HIV-negative and HIV-positive participants. Future studies may want to focus on mobile phone app design related to prevention and treatment separately in order to clarify how to best leverage technology for HIV prevention versus treatment. While focus group methodology enables interaction between participants, it is difficult to know if the recommendations and preferences that participants shared in a group accurately reflect their actual usage. The rapid pace of technological developments in mobile phones and social media make it difficult for research findings to keep up with usage in the real world. Future studies should pay particular attention to emerging technologies that may be leveraged for HIV prevention and treatment.

### Conclusions

As mobile technologies increasingly become tools for the prevention and treatment of HIV, these technologies should be integrated into existing models of care. Researchers would be wise to consider how technology can support BYMSM along the HIV prevention continuum and the HIV care continuum depending on serostatus. Our findings highlight interest in HIV prevention and treatment delivered via apps tailored for BYMSM, but raise significant concerns that must be addressed in order for them to be successful—namely privacy and a singular focus on HIV without attention to the other health and psychosocial issues impacting this population. It is crucial that public health practitioners partner with app developers to create apps that are engaging and provide a range of functions that meet the needs of BYMSM. Our research demonstrates the importance of including BYMSM in the development of these apps to ensure their effectiveness and uptake among this population disproportionately impacted by HIV.

## Abbreviations

AIDS: acquired immunodeficiency syndrome
ART: antiretroviral therapy
BYMSM: black young men who have sex with men
CFAR: UCLA Center for AIDS Research
CHIPTS: Center for HIV Identification, Prevention, and Treatment
CSTI: California Specialized Training Institute
GSN: geosocial networking
HIV: human immunodeficiency virus
HMP: HealthMpowerment
HOPE: Harnessing Online Peer Education
LA County: Los Angeles County
MSM: men who have sex with men
NIH: National Institues of Health
NIMH: National Institute of Mental Health
STD: sexually transmitted disease
STI: sexually transmitted infection
YMSM: young men who have sex with men
